# Economic Viability of Adoption of Automated Oestrus Detection Technologies on Dairy Farms: A Review

**DOI:** 10.3390/ani10071241

**Published:** 2020-07-21

**Authors:** Adewale Henry Adenuga, Claire Jack, Kehinde Oluseyi Olagunju, Austen Ashfield

**Affiliations:** Economics Research Branch, Agri-Food and Biosciences Institute (AFBI), 18a Newforge Lane, Belfast BT9 5PX, UK; claire.jack@afbini.gov.uk (C.J.); kehinde-oluseyi.olagunju@afbini.gov.uk (K.O.O.); austen.ashfield@afbini.gov.uk (A.A.)

**Keywords:** automated oestrus detection, investment, dairy, pedometer, accelerometer, precision agriculture, economic performance

## Abstract

**Simple Summary:**

The accurate and timely detection of oestrus is a central element of good dairy herd management as it ultimately determines the level of milk production and is core to the economic viability of the farm business. However, the traditional method of oestrus detection, which occurs by observing the dairy cows standing immobile while being mounted, is usually time-consuming, repetitive and requires considerable skill and experience on the part of the farmer to attain a reasonable level of efficiency. Given the limitation of the traditional method of oestrus detection, a number of automated oestrus detection (AOD) technologies have been developed. However, the rate of adoption of these technologies remains low. One reason that has been proposed for farmers’ low adoption of such technologies has been their lack of knowledge around the potential economic returns from investing in AOD technologies. In this paper, we review the empirical literature on the viability of investment in AOD technologies from an economic perspective. The conclusion of this study provides evidence from which farmers can make more informed decisions in relation to investing in AOD technologies. The review and analysis is also of importance for informing policy, as it provides an examination of the incentives and levers that could improve productivity on dairy farms.

**Abstract:**

The decision for dairy farmers to invest in automated oestrus detection (AOD) technologies involves the weighing up of the costs and benefits of implementation. In this paper, through a review of the existing literature, we examine the impacts of investment in AOD technologies in relation to the profitability and technical performance of dairy farms. Peer-reviewed articles published between 1970 and 2019 on the investment viability of AOD technologies were collated and analysed. We capture the different measures used in assessing the economic performance of investment in AOD technologies over time which include net present value (NPV), milk production, Benefit-Cost Ratio (BCR), internal rate of return (IRR) and payback period (PBP). The study concludes that investment in AOD technologies is not only worthwhile but also contributes to farm profitability.

## 1. Introduction

Reproductive management at farm level is a core element of dairy herd management and a central aspect of this is the accurate and timely detection of oestrus especially when artificial insemination (AI) is utilised [1,2]. The efficiency of oestrus detection on dairy farms has a direct relationship to improved insemination results, total pregnancy rate, as well as calf-to-conception intervals and this ultimately determines the level of milk production and consequently the economic success of the farm [1,2,3,4,5,6,7]. The traditional method of oestrus detection involved visual observation, which occurs by observing the dairy cows standing immobile while being mounted [8]. The method is, however, time-consuming and repetitive [1,9]. Oestrus detection requires cows to be watched at least three times a day, for a period of 20–30 min [7,10,11,12]. It therefore requires not only a lot of farm labour and time input but also considerable knowledge, skill and experience on the part of the farmer in order to attain a reasonable level of efficiency [5,13].

The inefficiencies in oestrus detection on dairy farms can be attributed to both farm-management- and cow-related factors. Specifically, about 10 per cent of the reasons for oestrus detection failure can be attributed to the latter, such as, anovular conditions and variable signs of oestrus with small numbers of cows expressing standing oestrus signs at any given time. However, the remaining 90 per cent is attributed to the former, that is, management and other external factors, which might be beyond the control of the farmer. These include the type of housing system provided for the cows and the accuracy of cow observation, the amount of time spent and the skill of the farmer and potentially the contribution of extreme weather conditions [14,15,16]. Generally, the increase in the cost of labour, herd size and workloads over the last decades has resulted in a decrease in the time available for visual observation per animal [14,17].

The adoption of automated oestrus detection (AOD) technologies has been identified as having the potential to increase reproductive efficiency on dairy farms and to contribute to overcoming some of the limitations of the traditional visual oestrus detection methods [18,19,20,21]. AOD technologies have the potential to contribute to shorter calving-to-conception intervals, increase the number of productive years per cow, reduce the culling rate due to poor fertility, and, overall, increases the lifetime production of milk per cow [22]. Furthermore, research has indicated that the use of AOD technologies increases electronic record keeping opportunities and provides a higher level of accuracy, hence contributing to overall dairy production efficiency through improved use of labour and time resources [2,23,24]. They, therefore, provide greater opportunities to meet farm production goals [23,24,25,26,27]. In spite of its advantages, the level of adoption of the modern (AOD) technologies on dairy farms remains low [28]. For example, research undertaken in the Netherlands indicates that only 20 per cent of Dutch dairy farms use AOD technologies [13]. Similar findings were reported for the province of Cremona in Italy, where only between 29 and 35 per cent of the dairy farmers employ AOD technologies [29].

A range of technical, personal, and economic factors have been identified as contributing to the low adoption rate of AOD technologies on dairy farms. The technical factors relate to the risks and uncertainties associated with its use, especially in relation to the lack of technical know-how and lack of confidence in the use of AOD technologies [30]. In terms of the personal factors, farmers tend to maintain a working relationship with the dairy cows such that they are more inclined to employ the traditional visual oestrus detection methods. Lastly, from an economic point of view, is the fact that farmers tend to be risk averse, especially in the absence of relevant information to support their decision making [31]. Lack of knowledge around the economic returns from investing in such new technologies (in terms of cost-to-benefit ratio and uncertainty in payback period to recoup investment cost), limits the adoption of the AOD technologies [24,28,32]. Indeed, research has actually shown that risk aversion is negatively correlated with the adoption of innovative technologies [31].

While there has been a number of review papers on the technical aspects of AOD technologies, for example Firk, Stamer, Junge and Krieter [7], Rorie, et al. [33], Galon [34], Saint-Dizier and Chastant-Maillard [17], and Reith and Hoy [5], to the best of our knowledge none have exclusively focused on the economic viability of investment in AOD technologies. Although a clear understanding of the workings of the AOD technology is essential from a theoretical point of view, translating that understanding to adoption of the technology by farmers is not always straight forward. This is because the farmers have to be convinced of its application to their particular farming system and that investment in the technology contributes to their goals of production efficiency and profit maximization. To fill this gap in the literature, this study reviews the existing empirical literature on the economic viability of investment in AOD technologies on dairy farms to provide evidence around investment in AOD technologies. In addition, our review also highlights the factors to be considered before investment in AOD technologies. Given that farmers usually adopt a technology on the basis of expected economic benefits, the results from this study will serve as a guide to assist farmers to make more informed investment decisions regarding AOD technologies. It also provides a firm research evidence base to inform policy makers engaged in designing policies that provide incentives and levers to improve farm level productivity.

## 2. Overview of AOD Technologies

Given the challenges associated with the traditional oestrus detection method and considering the impact of artificial insemination (AI) on reproductive performance of dairy cows, a number of AOD technologies have been developed and marketed in recent years to improve and automate the detection of oestrus on dairy farms [35]. These technologies, which can otherwise be described as precision dairy monitoring technologies, are designed to provide continuous surveillance of behaviour in the absence of, or in addition to, visual observation of oestrus [9,13,36]. AOD technologies are able to detect even slight changes in activity both during the day and at night, and often provide a recommendation for the optimal insemination time [22]. The majority of the technologies work by making use of software specific algorithms (sets of rules to follow during calculations) to compare a cow’s current behaviour with a cow’s specific reference period or in some cases, with the average activity of the herd aggregated over time, creating an oestrus alert when a set threshold is exceeded [15,17,20,22]. Senger [20] provides a summary of the functions of an ideal system for detecting oestrus, including: (1) provision of continuous and accurate surveillance of the individual cows in oestrus; (2) possession of a high level of efficiency and accuracy for identifying the appropriate physiological events necessary for predicting the timing of ovulation such that cows are inseminated at the correct time in relation to ovulation; (3) ensuring minimal labour requirements and; (4) ability to operate over the productive lifetime of the cow. Common examples of AOD technologies include activity meters or pedometry, temperature measurements, radio telemetric devices that monitor mounting activity, cameras, impedance or conductivity measurements, and hormone analyses [1,16,37,38]. These devices can be used either individually or in combination [7,39]. Generally, these AOD technologies detect oestrus in cows mainly through some kind of secondary signs of oestrus behaviour [40]. Examples of the secondary signs of oestrus include changes in physical activity, changes in electrical and chemical properties of reproductive tract secretions or mounting activity [17,26,33,39,40,41]. Studies by Van Eerdenburg, et al. [42] and Roelofs, van Eerdenburg, Soede and Kemp [1], have both shown that secondary signs of oestrus constitute as high as 63 per cent and 42 per cent of oestrus periods compared to standing oestrus, respectively. Similarly, a study by Mayo, Silvia, Ray, Jones, Stone, Tsai, Clark, Bewley and Heersche [9] shows that only 51 of the 109 cows (47%) stood to be mounted during visual observation, thus highlighting the importance of the secondary signs of oestrus relative to the traditional standing oestrus.

Although a variety of AOD technologies have been developed, pedometers and accelerometers clearly fulfil most of the criteria identified by Senger [20] and are the two most widely studied AOD technologies in the literature [26]. Given this, our review will focus specifically on these two technologies.

### 2.1. Pedometer

The pedometer is an electronic device that operates by transmitting information relating to the number of steps that the cow takes over a set time working, on the premise that oestrus in a cow is accompanied by increased physical activity—cows that are in oestrus do two to four times more walking than a non-oestrus cow [43,44]. Pedometers are usually placed on the neck (which records neck movements in all three dimensions), on the hind leg or on the front leg (which records the number of steps made by the cow per time unit). They can be used as stand-alone devices or be integrated into electronic animal identification systems to monitor cow movements. Cows coming into oestrus are identified by an increase in locomotion above the mean activity value recorded—during the same time period—for preceding days [15,44,45]. The data are usually recorded in 2-h increments, which are retrieved twice daily by an electronic scanner that is usually placed near the milking system. Data are then sent to the herd management software installed on the on-farm computer, which enables herd managers to review the reproductive status of individual cows [1,46]. An oestrus event is recorded if a cow’s weighted activity exceeds a user-defined threshold value relative to the cow’s baseline activity [1,44,46,47].

### 2.2. Accelerometers

Accelerometers measure acceleration in three special dimensions to assess changes in physical activity associated with oestrus [48]. “The common operation principle of accelerometers is based on a mechanical sensing element which consists of a proof mass (or seismic mass) attached to a mechanical suspension system with respect to a reference frame. Inertial force due to acceleration or gravity will cause the proof mass to deflect according to Newton’s Second Law. The acceleration can be measured electrically with the physical changes in displacement of the proof mass with respect to the reference frame” [49]. The device is able to measure the animal energy expenditure [50,51] travel speed [52], activity and feeding behaviour [53,54,55]. Accelerometers are usually attached to the neck collar, leg, or ear and estimate the overall activity making use of three-dimensional accelerometer technology [17,40,46]. They work by measuring continuously horizontal accelerations related to upward movements of the cow’s head and neck during walking and mounting behaviour [56]. The individual cow’s activity is continuously recorded by an accelerator sensor which calculates a general activity index in ‘activity units’ [56,57]. The data collected by the accelerometer is read by a transceiver unit and is automatically transferred on a real-time basis to the accelerometer herd management software installed on the on-farm computer. Data can be stored in 1-h or 2-h intervals. The raw activity data are analysed in a microprocessor by specifically developed, complex, mathematical algorithms, which calculate the weighted activity index based on deviations of the current measured data from the stored activity pattern within a specific period of time, thereby separating the cow’s day-to-day activity from activities associated with oestrous behaviour [46,56,58]. A list of cows determined by the accelerometer system to be eligible for insemination is then generated, and cows appearing on the list are subsequently inseminated by the farmer [16,46].

## 3. Methodology

Our review focused mainly on the pedometer and accelerometer oestrus detection technologies. These two technologies have been selected because they are the most studied AOD technologies in the literature. In addition, these two technologies, although requiring a relatively high initial cost outlay compared to other oestrus detection devices, have been found to provide the best results in the detection of oestrus in dairy cows, with a sufficiently high detection rate of between 80–90 per cent [7]. Given that this is a literature review, it was important to examine most of the available studies in this area. The articles used for the review were obtained from peer-reviewed journals, books and conference proceedings published between 1970 and 2019 in various databases. The length of years was chosen to be able to capture as wide range of articles as possible. The range of keywords and phrases used to search each database included: ‘pedometers’, ‘accelerometers’, ‘cow oestrus detection transponders’, ‘cow oestrus detection system’, ‘automated oestrus detection systems’, ‘profitability of automated oestrus detection systems’, and ‘economic impact of automated oestrus detection systems on dairy farms’. This was done systematically by focusing attention on a combination of the words and phrases. An example of a search combination of words used on SCOPUS was: TITLE-ABS-KEY (‘economic impact’ AND ‘automated oestrus detection systems’ AND ‘dairy farms’). The databases searched included the Web of Science, Science Direct, Google Scholar, Wiley Online Database, and EBSCO. Subsequently, the full papers were retrieved through the websites of the respective journals. The parameters with which the viability of investment in AOD technologies were measured include: calving interval, milk production, profit per dairy cow, net present value (NPV), benefit–cost (B:C) ratio, internal rate of return (IRR) and discounted payback period (DPBP).

## 4. Returns to Investing in AOD Technologies

Table 1, Table 2 and Table 3 detail key findings from articles included in our review. Most of the papers reviewed have employed either quantitative (usually through the development of normative models, which are either deterministic or stochastic) or qualitative (usually making use of descriptive statistics) methods [13,18,59]. The results show that it is profitable to invest in AOD technologies with positive net present value (NPV), internal rate of returns (IRR) greater than the discount rate and a reasonable length of payback period (PBP) (Table 1). This implies that the present value of the net revenues during the lifetime of the AOD technology exceeds the initial outlay of the investment. In addition, the studies also showed increased profit per cow (Table 1). Depending on the type of AOD technology, most of the studies showed that the payback period would be between three and half and eight years. For example, Giordano [60] found that given a tag cost of $120, an AOD system must remain functional for at least 5 years to break even and could generate as much as $13/cow per year in extra profits when life expectancy is 7 years. In a 2020 study, by Pfeiffer, Gandorfer and Ettema [22], the authors carried out an economic evaluation through the simulation of Simmental herds; they obtained a positive net return (NR) over all scenarios in the range of +€7 to +€40 per cow per year for the Simmental breed, and from +€19 to +€46 per cow per year for the Holstein Friesian breed. It is, however, acknowledged in some of the studies that to achieve maximal productive performance, AOD technologies should be combined with timed artificial insemination programmes (TAI) [60]. Of the studies reviewed, the PBP and profit per cow tend to be shorter and higher respectively for dairy farms in the US compared to other countries. For example, Dolecheck [47] obtained a PBP as short as 3.5 years and a profit margin ranging between $55.80 and $94.30 per cow. This may be attributed to the relatively more intensive dairy production system operated in the US compared to other countries. It should be noted that virtually all of the reviewed studies have employed a simulation approach except for Rutten, Steeneveld, Inchaisri and Hogeveen [18], which combined an experimental approach with a simulation model. While the general objective of all these studies were to analyse the economics of investment in AOD technologies, it is important to state that the scope of these studies in terms of parameters of interest varies. This is understandable given that dairy production systems and production characteristics are relatively different across the countries.

The effect of the use of the AOD technologies on other dairy production parameters is shown in Table 2. Only the parameters estimated in each of the studies have been included. The majority of the reviewed studies found that the use of the technologies leads to a reduction in calving interval. This is a very important finding, as it implies an improvement in milk production per dairy cow.

## 5. Cost of Investing in AOD Technologies

To adopt AOD technologies, farmers incur both variable costs (tag price) and fixed costs (initial investment cost), both of which exert varying influence on the investment results [47]. Our review of previous studies indicates that, although contributing to farm-level profitability, installation of AOD technologies on dairy farms can be relatively expensive. A summary of the cost of installation of the technologies on dairy farms across different countries is shown in Table 3. The results highlight that, in common with other capital investment, choosing to implement AOD technologies on farms incurs substantial upfront financial investment to cover the purchase, installation, and maintenance of the technologies. The level of investment profitability is usually influenced by a number of factors, including the price of milk (high), the size of the herd (large) or the percentage of cows equipped, the oestrus-detection rate and culling rate, and the time when the investment is made [22]. For example, with large herd size, farmers are more likely to require more labour to undertake the traditional visual oestrus detection, which can be expensive. In such a situation, it is optimal to invest in AOD technology to reduce costs of labour and consequently maximize profit. The implication of this is that the return on investment can be different for different farms, depending on the farm characteristics [18]. The relatively high cost of investing in the AOD technology also contributes to its low level of adoption by farmers. For example, a study by Neves and LeBlanc [32] using a survey-based approach showed that the majority of dairy farmers without AOD technology on their farms stated that the high cost-to-benefit ratio of the technology was the reason for their non-adoption.

## 6. Factors Influencing the Decision to Invest in AOD Technologies

To invest in AOD technologies, farmers need to know what factors have to be considered. In this section, we outline those factors that need to be taken into account by farm managers to make an informed decision to invest in AOD technology.

### 6.1. Oestrus Detection Rates of the Technology AOD

While the use of AOD technologies generally results in better detection of oestrus in dairy farms compared to visual observation, it should be noted that the sensitivity of the technology varies across product types, and this is an important factor that must be considered in the decision to employ the AOD technology in dairy farms. In fact, a study by Dolecheck [47] has shown that the oestrus detection rate of AOD technologies have the greatest effect on NPV compared to the cost of the investment. Oestrus detection rate using activity meters generally ranges between 59 and 92 per cent in the literature [7,22]. When the sensitivity of the AOD technology is high, it results in the shortening of the average calving interval, which consequently increases annual milk production accordingly and culling of fewer cows for failing to conceive [22]. However, an increase in sensitivity has a stronger effect on the average calving interval at low sensitivities than at high sensitivities [18]. The implication of this is that the potential impact of the adoption of AOD technologies depends on the pre-existing fertility management of a dairy farm. Generally, dairy farms that initially have a high visual oestrus detection rate and high labour input, tend to have little or no financial benefit from their adoption of AOD technology—although, the use of the technology may lead to a larger saving in labour input. On the other hand, dairy farms with previously poor visual oestrus detection rates and a low labor input are likely to benefit more financially from the adoption of AOD technologies [18,22]. For example, in the study by Rutten, Steeneveld, Inchaisri and Hogeveen [18], they found that increasing the sensitivity from 30 to 40 per cent shortened the average calving interval by 7 days, from 435 to 426 days, whereas increasing the sensitivity from 85 to 95 per cent shortened the average calving interval by only two days, from 401 to 399 days. In addition to the sensitivity of the AOD technology, Giordano [60] has also found that for investment in AOD technology to be profitable, the life expectancy of the technology must be at least five years. This is in view of the fact that a longer life expectancy of the system significantly reduced the fixed costs of purchase and installation [63]. (It should be noted, however, that the lifespan of the technology might not be determined only by the quality of the product but also the care and maintenance provided by the farm).

### 6.2. Labour Costs

Labour costs are an important factor to be considered in the decision to invest in AOD technologies [19,64]. This is because the visual oestrus detection method requires more labour hours [7]. Arendzen and Scheppingen [64] estimated that investment in AOD technologies could reduce labour costs by 30 per cent. This is particularly important in situations where labour costs are high as is currently the case in Northwest Europe due to declining labour availability for agricultural production [65]. It is, however, important to note that in situations where the labour cost is relatively low, investment in AOD technologies may not be viable [66]. For example, in the study by [67], farms that invested in AOD technologies, but incurred large fixed costs including contractor costs and costs for gas, water, and electricity, etc., had lesser profit than farms using the conventional oestrus detection method, even though this required the use of more labour.

### 6.3. Environmental Conditions

In considering whether to invest in AOD technology, another important factor that should be considered is the type of housing and associated environmental conditions to which the dairy cows are subjected. Generally, the AOD technologies are likely to be more effective for cows kept in free stall barns compared to those kept in tied stalls [7]. For example, a study by [68] has shown obvious differences in percentage increase of activity in oestrus between cows kept in free stall barns and those kept in tied stalls. Indications were that cows kept in free-stalls showed greater levels of activity compared to those kept in tied stalls. Studies have also shown that the performance of pedometers and activity meters for oestrus detection are influenced by the environmental conditions (production and feeding strategies, oestrus detection and culling strategies) to which the cows are subjected [45,69]. For example, oestrus behaviour of cows is influenced by the substrate of the floor on which they are housed [70,71]. As a result, AOD technologies are likely to function more efficiently when cows are housed on soft surfaces compared to concrete surfaces [5,72]. Britt, Scott, Armstrong and Whitacre [71] have also shown that the duration of oestrus activity was reduced by about 25% when cows were kept on concrete as opposed to softer underfoot conditions. In situations where a concrete floor has to be used, it should be a concrete slatted floor covered with perforated rubber mats. Roelofs, et al. [73], in contrast, has shown that the performance of activity meters to detect oestrus is not affected by housing conditions, whether on pasture or indoors. Another environmental factor that could affect the efficiency of AOD technologies is stress on the part of the animal. Schüller, et al. [74] showed that during long- and short-term stress, the use of pedometers resulted in lower conception rate as a result of the stress, such that cows were between 63% and 80% less likely to get pregnant independent of the type of semen employed compared with animals without stress. Another factor that should be considered is the method of calving employed in the calving herd. The study by Bekara, Bareille, Bidan, Allain and Disenhaus [61] has shown that the use of AOD technology to increase the rate of oestrus detection had a significant effect on calving interval for herds with continuous calving (reduction of between 10 and 23 days) compared to herds with grouped calving (reduction of between 6 and 11 days). It was explained in the study that the reason for the differential impact is linked to the higher culling rate of cows with reproductive disorders or infertility in herds with grouped calving than in herds with continuous calving [61]. Consequently, the improvement in the sensitivity of heat detection has a greater impact on herds with spread calving.

### 6.4. Herd Size and Milk Price

The size of the herd is also an important factor to be considered in the decision to invest in the use of AOD technologies on dairy farms [75,76]. The effect of herd size corresponds to a reduction in the purchase price of a data transmission system and software, which is fixed regardless of the size of the herd [77]. This assertion is supported by Abeni, Petrera and Galli [29] in which they found that the percentage of farmers using at least one sensor for AOD was greater in farms with larger herds than in those with smaller herds. While about 62 percent of farms with greater than 201 cows were using pedometers, it was only about 10 percent for farms with fewer than 101 cows. This is further supported by Gargiulo, Eastwood, Garcia and Lyons [77] who found that farms with larger numbers of dairy cows may benefit more from the introduction of automated systems to monitor individual cows compared to farms with smaller numbers of dairy cows. Just like the conventional method of oestrus detection, the efficiency of AOD technologies is influenced by the fact that the intensity of expression of oestrus in dairy cows is higher when they are in large groups. This results from the fact that the expression of oestrus behaviour by cows results in the sexual stimulation of others, such that an increasing number of animals are in oestrus at the same time [1,42,45].

The returns on investment on AOD and profitability of dairy production depends significantly on milk price. When the price of milk is high, it is easier for farmers to cover the costs of investment in AOD technologies [61]. The improvement in the sensitivity of oestrus detection increases the quantity of milk produced per cow [18] and consequently, the profitability of dairy production.

### 6.5. Data Management Proficiency

Good records not only contribute to good farm management practice but are an essential step in all infertility investigations. Proficiency in data management is an important factor that should be considered when making a decision whether to invest in AOD technologies. According to [78,79], the ability to be able to make good use of the output data from AOD technologies is more important than having the technology itself. This is because the technologies themselves do not automatically improve production performance. This issue can be reduced by ensuring that the AOD technology has a user-friendly and time-saving interface for the digital control of the technology [80]. This implies that it might be necessary for farmers to acquire the necessary skills for technology management before taking the decision to invest in the technology [29]. This assertion is supported by Bewley, Russell, Dolecheck, Borchers, Stone, Wadsworth, Mayo and Tsai [59], who reported that about 55 per cent and 42 per cent of sampled dairy farmers in their study perceived that a lack of familiarity with the available AOD technologies and high cost-to-benefit ratio, respectively, as limiting factors to the use of AOD technology.

## 7. Conclusions

This paper has reviewed the economic consequences of investment in AOD technologies and also its effect on technical parameters of the farm such as milk production. We found that investment in AOD technologies is economically viable for most dairy farms. However, the level of awareness of the significance of adoption of AOD technologies is still relatively low given the current level of adoption of the technology. The paper also shows that AOD technologies contribute to the improvement in economic and technical performance of dairy farms. Furthermore, the full economic value of AOD technologies is difficult to determine because they can provide additional benefits (e.g., health monitoring and cow comfort evaluation) beyond oestrus detection, which can result in additional cost savings. AOD also has the tendency of contributing to more timely and informed decisions and, consequently, a higher profitability for the farm. This highlights a need to better inform farmers of the financial and production benefits of investing in AOD technologies. This review will be useful to famers in helping them make decisions about investing in AOD technologies. It will also assist policy makers and the government in making decisions around what type of AOD technologies to recommend to farmers and also to justify the need to support farmers’ adoption of AOD technologies.

In making use of the conclusion from this study in decision making, it is important to take into consideration the fact that different methodologies have been employed in the papers reviewed and different market conditions, which might affect the viability of investment in the technologies in the different countries. Specifically, the majority of the studies employed simulation techniques, such that the results from the investment analyses are highly dependent on the assumptions employed in the models, especially in relation to oestrus detection rate, the number of pregnancies per artificial insemination and labour costs. Further, it should be noted that the adoption of AOD technologies alone in itself does not guarantee efficiency of the system. In conditions where the oestrus detection rate is already high with the use of the traditional visual oestrus detection technique, for example, above 70 per cent, or when the cost of labour is relatively low, the use of AOD technologies may not be profitable. Other factors such as accurate record keeping of activities on the farm in an organised and structured way, cow and environmental factors, including the type of housing and the herd size, all contribute to the efficiency of the system. A combination of techniques can also be employed to optimize the reproductive management systems on dairy farms.

The countries identified in this paper are the few countries where studies relating to the economic viability of adoption of automated oestrus detection technologies on dairy farms have been undertaken, and our literature search was undertaken without any emphasis on particular group of countries. This emphasises the need for more research to be done on the economic consequences of investments in precision technologies in general, and on the factors influencing their adoption in dairy farms. For example, it would be interesting to see an increase in the number of published papers in this area that employ econometric techniques to analyse the factors influencing the adoption of automated oestrus technologies and the efficiency of the system.

## Figures and Tables

**Table 1:** Economics of investment in automated oestrus detection (AOD) technologies for different dairy farm types.

References	Country	Type of AOD	^1^ NPV(DR)	^2^ IRR (%)	^3^ PBP (yrs)	Profit (Per Cow Per Year)	Study Methodologies
Bekara, et al. [61]	France	Activity meter and pedometer	Positive (2.1%)	0.04 to 33.8	NF	+€8.5 to +€ 92	Simulation model
Rutten, Steeneveld, Inchaisri and Hogeveen [18]	The Netherlands	Activity meter	Positive (5%)	11	8	€21.74	Simulation model
Dolecheck [47] ** a	US	AOD	Positive		3.8	$55.80 to $94.30	Simulation model
Dolecheck [47] ** b	US	AOD	Positive	NF	3.5	$55.80 to $94.30	Simulation Model
^4^ Fricke, Giordano, Valenza, Lopes, Amundson and Carvalho [41]	US	Activity meter	Positive	NF	NF	NF	Experimental combined with simulation model
Giordano [60]	Northeast U.S.	AAM systems Plus synchronization of and ovulation protocols		NF	7	$31	Simulation model
Pfeiffer, Gandorfer and Ettema [22]	Germany	Activity meter	Positive	NF	7	+€7 to +€40 (Simmental breed) and +€19 to +€46 (Holstein Friesian breed)	Simulation model
^5^ van Asseldonk, et al. [62]	The Netherlands	Activity meter	NF	NF	NF	€43.2	Simulation model

^1^ The NPV (net present value) indicates whether the present value of the net revenues during the detector lifetime exceeds the initial outlay of the investment. It is calculated as the sum of all present values of revenues and all present values of expenses [61]. ^2^ The IRR (internal rate of return) is the discount rate value that nullifies the NPV. That is, the rate at which the NPV is zero. This percentage is an indicator of the annual return on invested capital over the lifetime of the investment. If the IRR is higher than the chosen discount rate, then the investment is profitable. ^3^ The PBP (payback period) is the number of years needed to pay back the initial purchase costs, which is calculated using discounted cash flows. The PBP is defined as the year in which the sum of present cash flows equals or exceeds the initial purchase costs. ^4^ This study compared reproductive performance of cows managed using timed artificial insemination (TAI). ^5^ An exchange rate of Dfl. 1 = €0.45 has been applied to estimate the profit per cow per year. NF—this implies that the parameter is either not included or has not been estimated in the study. DR refers to the discount rate used in the study. ** This is the same study but employs different parameters.

**Table 2:** Effect of Investment in AOD Technologies on Dairy Production performance.

References	Calving Interval (Days)	Labour	* ODR (Sensitivity)	Milk Yield
Bekara, Bareille, Bidan, Allain and Disenhaus [61]	−7 to −23	NF	50% vs. 90%	5200 kg to 8450 kg
Rutten, Steeneveld, Inchaisri and Hogeveen [18]	−16	−4 h per week	50% vs. 80%	8310 kg
Dolecheck [47] ** a	−15.3	NF	48.6% vs. 60%	10,758 kg
Dolecheck [47] ** b	−35.3	NF	48.6% vs. 80%	10,758 kg
Giordano [60]	NF	NF	30% vs. 80%	12,700 kg
Pfeiffer, Gandorfer and Ettema [22]	NF	NF	55% vs. 92%	7000 and 11,000 kg ^a^
van Asseldonk, Jalvingh, Huirne and Dijkhuizen [62]	NF	NF	50% vs. 90%	7500 kg

* ODR = oestrus detection rate used in the models and it is the baseline against the assumed rate for AOD technologies. ^a^ Two different breeds were considered: Simmental herds with milk yields of 7000 or 9000 and Holstein Friesian herds with milk yields of 9000 or 11,000 kg/yr. NF—this implies that the parameter either is not included or has not been estimated in the study. ** This is the same study but employs different parameters.

**Table 3:** Cost of investment in AOD technologies.

References	Type of AOD	Cost of Sensor System Data Transmission System and Software Per Unit	Collar (*/Cow)	Annual Maintenance and Replacement Costs (*/Year)	Country
Bekara, Bareille, Bidan, Allain and Disenhaus [61]	Pedometer	€6498	€107	Guaranteed, not included in estimation	France
Bekara, Bareille, Bidan, Allain and Disenhaus [61]	Activity meter	€4430	€120	Guaranteed, not included in estimation	France
Rutten, Steeneveld, Inchaisri and Hogeveen [18]	Activity meter	€3600 per herd	€108	€90 per year	The Netherlands
Dolecheck [47]	AOD	$US5000	$US50	($US0)	US
Dolecheck [47]	AOD	$US10,000	$US100	($US0)	US
Giordano [60]	Activity meter	$US8000	$US120	$US1500 per year to account for maintenance and lost tags	US

* Monetary unit (€, US$).
